# Diversity of Phytochemical and Antioxidant Characteristics of Black Mulberry (*Morus nigra* L.) Fruits from Turkey

**DOI:** 10.3390/antiox11071339

**Published:** 2022-07-08

**Authors:** Sona Skrovankova, Sezai Ercisli, Gursel Ozkan, Gulce Ilhan, Halil Ibrahim Sagbas, Neva Karatas, Tunde Jurikova, Jiri Mlcek

**Affiliations:** 1 Department of Food Analysis and Chemistry, Faculty of Technology, Tomas Bata University in Zlin, 76001 Zlin, Czech Republic; mlcek@utb.cz; 2 Department of Horticulture, Faculty of Agriculture, Ataturk University, 25240 Erzurum, Turkey; sercisli@atauni.edu.tr (S.E.); gozkan08@atauni.edu.tr (G.O.); gulce.ilhan@atauni.edu.tr (G.I.); halil.sagbas@atauni.edu.tr (H.I.S.); 3 Department of Nutrition and Dietetics, Faculty of Health Sciences, Ataturk University, 25240 Erzurum, Turkey; ngungor@atauni.edu.tr; 4 Institute for Teacher Training, Faculty of Central European Studies, Constantine the Philosopher University in Nitra, 94974 Nitra, Slovakia; tjurikova@ukf.sk

**Keywords:** black mulberry, phytochemical assessments, total phenolics, total anthocyanins, antioxidant activity, DPPH and FRAP, flavonoids, phenolic acids, HPLC

## Abstract

Black mulberry (*Morus nigra* L.) fruits are known due to their delicious, sweet and slightly acid flavor and high anthocyanin content. In the present study, the diversity of phytochemical, sensory, and antioxidant characteristics of the fruits of 20 black mulberry genotypes, from the Artvin region of Turkey, were evaluated. As important phytochemical assessments in fruits, we chromatographically (HPLC/DAD) determined glucose (7.22 to 11.10 g/100 g fresh weight (fw)) and fructose content (6.32 and 9.94 g/100 g fw), as well as predominant organic acid in black mulberry genotypes fruits—malic acid (6.02–11.44 g/100 g fw), followed by citric acid. Titrative determination was used for ascorbic acid, finding contents of 17.41–28.33 mg/100 g fw. There was found a great diversity of sensory (taste, juiciness, and aroma) characteristics, indicating a richness of the fruit germplasm. Antioxidant parameters, such as total phenolic (TP) and anthocyanin (TA) content, were assessed spectrometrically; antioxidant activity (AA) was assessed by DPPH and FRAP assays; individual flavonoids and phenolic acids were determined chromatographically (HPLC/DAD). Antioxidant characteristics of the fruits, defined by TP and TA content, ranged from 1951 to 2733 μg GAE/g fw and 508–712 μg C3GE/g fw, respectively. The most abundant compounds of flavonoids and phenolic acids groups were determined to be rutin (47.10–97.20 mg/100 g fw) and chlorogenic acid (51.3–90.8 mg/100 g fw). AA results, measured by the DPPH method as EC_50_ value, ranged between 16.10 and 25.45 μg/mL; a FRAP assay revealed values of AA between 9.80 and 13.22 μmol TE/g fw. Significant differences in phytochemical and antioxidant qualities were observed among the analyzed *M. nigra* genotypes. Regarding the best values of phytochemical and antioxidant characteristics, three genotypes of *M. nigra* were selected to be recommended for fruit production. The results thus highlight the potential for the exploitation of local black mulberry genotypes through crop selection and breeding program.

## 1. Introduction

In recent years there has been a considerable increase in interest in fresh fruits, mainly due to changes in production trends, marketing, and effective advertisement on social media with regards to the abundant amount of bioactive compounds in fruits. Fruits can supply several nutrients to humans, and are important constituents of a healthy and well-balanced diet. Fruits are major sources of provitamins A and vitamin C, and constitute a great source of phytochemicals and bioactive components with the potential to be treatment agents for cardiovascular diseases and possibly reduce risk of cancer. Moreover, fruits provide fiber and minerals for a healthy adult diet, as well as diversity in flavors and colors. These characteristics, as well as the level of important bioactive compounds in fruits, have been shown to be greatly influenced by genotypes and environmental growth conditions [1,2,3,4].

In particular, berries enjoy the status of a ‘super-food’, with great interest in fruit species in nutritional science. It is also due their beneficial influence on human health [5,6,7]. From a phytochemical point of view, this is caused by the high content of anthocyanins and other phenolic substances in these fruits. Berry fruits are commonly consumed in fresh and frozen form as well as in processed products. [8,9,10,11,12].

Mulberries belong to the genus *Morus* of the family Moraceae, which originate from the area between India and China. It is believed that domestication of mulberry trees started several thousand years ago for silkworm rearing. They are adapted to tropical, subtropical, and temperate zones, naturally growing in a wide range of topographic and soil conditions, from sea level to altitudes of 4000 m. The plant has a high capacity for adaptation to various soil and environment conditions [13].

The mulberry tree is amenable to both sexual and vegetative propagation. Therefore, diverse levels of polyploidization in the genus are reported, with wide variation of chromosome numbers, such as 2n = x = 14 chromosomes in *M. notabilis*, 2n = 2x = 28 in *M. alba* and *M. indica*, 2n = 3x = 42 in *M. bombysis*, 2n = 4x = 56 in *M. laevigata, M. cathayana*, and *M. boninensis*, 2n = 6x = 84 in *M. serrata* and *M. tiliaefolia*, and 2n = 22x = 308 in *M. nigra* [13].

Black mulberry (*M. nigra*) is much more difficult to propagate than the white mulberry type, and grows much slower than white ones. Black mulberry trees are particularly abundant in the Aegean and Mediterranean regions, but are also well represented in other agricultural areas of Turkey, such as Marmara, inner Anatolia, eastern Anatolia, and the Black Sea regions. In Turkey, the majority of mulberry trees belong to the white mulberry (*Morus alba*) type. They were cultivated due to the high-yield fruit capacity of the trees (harvested several times in a year) and for the processing of special products used for molasses production [14]. In contrast, black mulberry fruits are not suitable for molasses making. However, black mulberry trees have gained importance due to their bigger, colorful, visually attractive fruits with a unique, slightly acidic flavor. Thus, they can be consumed as fresh fruits or processed as fruit juices [15]. Black mulberry fruits were traditionally used to produce various products, such as juices and jams, and were also used for centuries for medicinal purposes e.g., in a mouth lesion treatment [16]. At present, there is also a notable consumer demand for black mulberry fruits due to their great organoleptic characteristics and high content of phytochemicals [15,17]. The high concentration of anthocyanins found in black mulberry fruits have been proven to have an inhibitory effect on cancerous liver cells in human patients [18]. It has been reported that mulberry fruits and their products, such as molasses, juices, compotes, and jams, could help in the treatment of some coronary problems, as well as gastric and intestinal diseases [19]. Moreover, black mulberry trees are resistant to pests and various diseases. All these facts make its fruits suitable for the development of novel products with great value, particularly in the context of promoting a healthy, organic, and sustainable lifestyle [20].

In the Artvin region of Turkey, black mulberries are quite abundant, but no study has been performed on these exact genotypes. Thus, the main objective of this study was to provide a comprehensive characterization of local black mulberry genotypes, and to explore their diversity and potential for optimal crop selection and breeding programs.

## 2. Materials and Methods

### 2.1. Characterization of Analyzed Fruit Material

In the present study, 20 black mulberry (*M. nigra*) genotypes (VC1–VC20; VC means Valley of Coruh) were analyzed (Table 1). Black mulberry fruits were collected from 30 year old healthy trees in July 2018 (from 11 to 28 July). Trees were cultivated under the same pedoclimatic and cultural conditions in the Artvin province, located in the north-eastern part of Turkey. The climate in Artvin is characterized by mild winters and slightly hot and dry summers.

For fruit analysis, 1 kg of fruits per genotype was harvested and transported to the laboratory in a cold chain. Samples for analysis were prepared from the fresh berry form using a food blender (Heidolph Crusher M, Schwabach, Germany) and measured immediately after preparation, or from the thawed form after deep freezing (−80 °C). For each fruit sample and analysis, three replicates were prepared.

The weight of fruits was measured from 40 fruits per genotypes, with four replications (Table 1). Fruit weight was determined using an electronic balance (Mettler Toledo ME-T, Greifensee, Switzerland). Fruit width/length ratio was used to determine the fruit shape index. Crown habits were determined as either round (genotypes VC1, VC4, VC8, VC14, VC18, VC20) or half open (genotypes VC2, VC3, VC5–7, VC9–13, VC15–17, VC19).

### 2.2. Determination of Soluble Solid Content, pH, Titratable Acidity, and Maturity Index

For the determination of soluble solid content (SSC) (%), a digital refractometer (ATAGO PR-32, Atago, Saitama, Japan) was used; for pH measurements a pH meter (Mettler Toledo F20, Switzerland) was applied. Titratable acidity (TA) was measured potentiometrically by titrating the sample with 0.1 M NaOH until the pH reached value 8.1, and was expressed as % citric acid equivalent. Maturity index (MI) was expressed as a SSC/TA ratio.

### 2.3. Determination of Individual Sugars

For individual sugar (fructose, glucose, and saccharose) analyses, a modified method from Melgarejo et al. was used [21]. Homogenized fruits (5 g) were diluted with purified water. The homogenate was centrifuged at 6000 rpm for 5 min. Supernatants were filtered through a 0.45 µm membrane filter (Iwaki Glass, Sumedang, Indonesia) before the analysis. The HPLC analysis was conducted using a PerkinElmer HPLC system (Waltham, MA, USA) with an Amino NH2 column (Waters, Milford, MA, USA) and 85% acetonitrile/15% H_2_O (*v*/*v*) as a mobile phase. A refractive index detector (RID) was used. Samples were identified and quantified by standards. Results were expressed as g/100 g fruit weight (fw).

To specify the sweetness perception of the 20 fruit genotypes, their sweetness indices (*SI*) were calculated using the methods of Roussos et al. [12]. The *SI* index considers the relative sweetness as a factor of each of the three sugars measured. It is described in the following Equation (1):

(1) SI=1.00  Glu+2.3  Fru+1.35  Sacch

where *Glu* stands for glucose concentration, *Fru* for fructose concentration, and *Sacch* stands for saccharose concentration.

### 2.4. Determination of Organic Acids

Organic acids were determined by the Bevilacqua and Califano method [22]. To berries (10 g), a solution of 0.009 N H_2_SO_4_ (10 mL) was added and homogenized (Heidolph Crusher M, Schwabach, Germany). The samples were then centrifuged for 15 min at 14,000 rpm. The supernatants were filtered through filter paper and a 0.45 μm membrane filter (Millipore Millex-HV Hydrophilic PVDF, Burlington, MA, USA), and finally a SEP-PAK C18 cartridge. Organic acids were determined by HPLC (Agilent 1100 series HPLC G 1322 A, Waldbronn, Germany) using an Aminex column (HPX-87H, 300 mm × 7.8 mm, Bio-Rad, Hercules, CA, USA) with mobile phase 0.009 N H_2_SO_4_. Organic acids were detected at wavelengths 214 and 280 nm. Samples were identified and quantified by standards. The results were expressed as g/100 g fw.

### 2.5. Sensory Analysis of Fruits

For the sensory analysis of the fruit, the methodology developed at Ataturk University was used [23]. A trained panel of five experts evaluated three sensory features (taste, juiciness, and aroma) of black mulberry fruits for each genotype. The 0 to 9 bipolar hedonic scale was used to rate overall pleasantness of taste, juiciness, and aroma, which were rated on a unipolar 0 to 9 intensity scale. For taste, the scale indicated 0 = not detectable; 1 = extremely sour; 3 = sour; 5 = sweet–sour; 7 = sweet; and 9 = extremely sweet. For juiciness, the scale indicated 0 = not detectable; 1 = extremely low; 3 = low; 5 = medium; 7 = high; and 9 = extremely high. For aroma, it was indicated as 0 = not detectable; 1 = just barely detectable; 3 = slight; 5 = moderate; 7 = intense; and 9 = extremely intense. Afterwards the most evaluated characteristic was defined.

### 2.6. Determination of Ascorbic Acid (Vitamin C), Total Phenolic, and Total Anthocyanin Content

Homogenized fruits (5 g) were used for ascorbic acid (vitamin C) analysis. Its content was determined using the AOAC method [24], by titration with 2,6-dichlorphenolindophenol sodium salt solution using chloroform for intensely colored extracts.

Fruit phenolics were extracted from fruits (10 g) using 80% ethanol (40 mL) in water bath (80 °C), kept for 20 min in inert atmosphere, and filtered through Whatman filter paper. Extraction of the residue was repeated under the same conditions. The filtrates were combined and diluted with 80% ethanol to 100 mL, and the obtained extract was used for the determination. The quantification fruit phenolics was determined by the Folin–Ciocalteu method [25], with some modifications. An total of 1 mL of extract with 20 mL of deionized water and 1 mL of Folin–Ciocalteu’s reagent was mixed. After 3 min, 5 mL 20% Na_2_CO_3_ was added, then replenished with deionized water to a volume of 50 mL and mixed. After 30 min incubation at room temperature, the absorbance at 765 nm was determined by a UV-Mini 1240 spectrophotometer (Shimadzu, Kyoto, Japan). Results were expressed to standard, gallic acid, as μg gallic acid equivalent (GAE)/g of fw.

The total anthocyanin content of fruits was determined using the bisulphite bleaching method [26]. Anthocyanins were extracted from 2 g of fruits with 0.1% HCl (2 mL) in 96% ethanol and 2% HCl (40 mL). The mixture was centrifuged at 5500 rpm for 10 min. The mixture for measurement: extract (10 mL) and 15% sodium bisulphite (4 mL). After 15 min of reaction at room temperature, the absorbance was measured at 520 nm by a UV-Mini 1240 spectrophotometer (Shimadzu, Kyoto, Japan). The molar absorbance value for cyanidin-3-glucoside (C3GE) was used as a standard. Results were expressed as μg of cyanidin-3-glucoside equivalents/g of fw.

### 2.7. Determination of Phenolic Acids and Flavonoids

The HPLC technique was employed for the determination of phenolic compounds by modification of the method by Rodriguez-Delgado et al. [27]. An amount of 5 g from each homogenized sample was diluted with distilled water (1:1) and centrifuged at 15,000 rpm for 15 min. The supernatants were filtered through a 0.45 µm membrane filter (Millipore Millex-HV Hydrophilic PVDF, Burlington, MA, USA). HPLC analysis was realized by Agilent 1100 series HPLC equipment, with ODS column (250 mm × 4.6 mm, 4 µm (HiChrom, Chadds Ford, PA, USA)) and mobile phase composed of Solvent A (methanol/acetic acid/water (10:2:88, *v*/*v*/*v*)) and Solvent B (methanol/acetic acid/water (90:2:8, *v*/*v*/*v*) with a gradient program. For the identification of chromatographic data, a DAD detector (Agilent, Santa Clara, CA, USA) was used, with spectral determination at 254 and 280 nm. Samples were identified and quantified by standards. The results were expressed as mg/100 g fw.

### 2.8. Determination of Antioxidant Activity

Antioxidant activity was determined with two methods—the DPPH (2,2-diphenyl-1-picrylhydrazyl) free radical scavenging test, and a FRAP (Ferric Reducing Antioxidant Power) assay.

The DPPH assay was performed spectrometrically, according to the modified method of Brand-Williams et al. [28]. Homogenized fruits (5 g) were extracted with methanol, and, after filtration (filtration paper), solutions of the fruit extracts were prepared in the following concentrations: 0.02, 0.04, 0.06, 0.08, and 0.1 mg/mL. To each prepared solution (2 mL), 0.5 mL of 1 mM DPPH (Sigma-Aldrich, St. Louis, MO, USA) solution in methanol was added. After incubation for 15 min at room temperature, the absorbance was measured at 517 nm against blank solution (containing methanol in same amount as solutions volume and DPPH solution). The results were then expressed as half of maximal effective concentration (EC_50_) (μg/mL).

The FRAP assay is based on the reduction of ferric-2,4,6-tripyridyl-S-triazine (Fe(III)-TPTZ) complex into the blue-colored Fe(II) form in the presence of antioxidants, and was performed according to the modified method of Benzie and Strain [29]. Homogenized fruits (5 g) were extracted with methanol, and after filtration (filtration paper) were used for the FRAP test. A total of 1 mL of the extracts was mixed with freshly prepared FRAP solution (1.5 mL) containing 25 mL of 300 mM acetate buffer (pH 3.6), 2.5 mL of 10 mM 2,4,6-tripyridyl-s-triazine (TPTZ) in 40 mM HCl solution, and 2.5 mL of 20 mM ferric chloride (FeCl_3_·6H_2_O) solution. The absorbance of present blue color, indicating the ability of the sample to reduce the ferric tripyridyltriazine (Fe(III)-TPTZ) complex, was measured at 593 nm. Results were expressed against standard Trolox (TE, 6-hydroxy-2,5,7,8-tetramethylchromane-2-carboxylic acid) in Trolox equivalents, μmol TE/g fw.

### 2.9. Statistical Analysis

All data were analyzed using SPSS software and procedures. Analysis of variance tables were constructed using the Least Significant Difference (LSD) method at *p* ≤ 0.05 (IBM SPSS Statistics for Windows, Version 26.0, Armonk, NY, USA, 2019). The principal coordinate analysis (PCoA) was performed to show the relationships and differentiation of the morphological and biochemical traits of black mulberry genotypes in a three-dimensional array of eigenvectors using the DCENTER and EIGEN modules of NTSYS-pc 2.10e software (Numerical Taxonomy and Multivariate Analysis System, Version 2.1, Exeter, Brookhaven, NY, USA, 2000).

## 3. Results and Discussion

### 3.1. SSC, pH, Titratable Acidity, and Maturity Index

The values of the SSC, pH, titratable acidity, and maturation indices of 20 black mulberry genotypes are shown in Table S1. Significant differences (*p* ≤ 0.05) among fruit genotypes were observed for SSC and pH values. However, no significant differences for the titratable acidity of the analyzed genotypes were detected. There are many factors that are included in the term of fruit quality. Fruit size (weight), fruit firmness, soluble solids, and titratable acidity are the most important for many fruits.

The SSC content of black mulberry fruits in this study ranged between 13.36% and 17.95%, with an average of 15.79%. These results are similar to the research of Elmaci and Altug [30], who studied three black mulberry genotypes (11.3–16.2%) in Turkey. A study performed by Okatan [20] exhibited similar SSC data for 13 native black mulberry genotypes grown in the western region of Turkey (14.23–19.43%), as did research by Koyuncu [31] on 28 native black mulberry genotypes from the Mediterranean region in Turkey (SSC between 13.11 and 16.23%) and a study by Ercisli et al. [32] on four Turkish fruit genotypes (16.95–18.40%). In addition, work produced by Calín-Sánchez et al. [33], who studied four Spain genotypes, showed similar SSC results, between 12.0 and 25.8%, as did research by Okatan et al. [34] that analyzed eight black mulberry genotypes grown in eastern Anatolia, with an observed range from 15.6 to 22.1%.

Assessed pH values of the fruits analyzed in the present study were between 3.55 and 4.12, which is similar to values measured by Okatan et al. [34], who analyzed eight black mulberry genotypes grown in eastern Anatolia with observed pH values of 3.65–4.12. Elmaci and Altug [30] found similar values (pH 3.60–3.80), as did Okatan [20], with pH results ranging from 3.66 to 4.42, and Koyuncu [31], in a study that involved 28 native black mulberry genotypes from the Mediterranean region in Turkey, with pH values of 3.22–3.47. However, these data are lower to those presented by Calín-Sánchez et al. [33] for Spanish fruits (pH 5.95–7.39). Results of titratable acidity ranged from 1.38 to 1.87% citric acid equivalents, with no relevant difference to the results of Elmaci and Altug [30] (1.51–1.79%), Okatan [20] (1.47–1.93%), Okatan et al. [34], who analyzed eight black mulberry genotypes (1.45% to 1.85%), Ercisli et al. [32] (1.64–1.97%), or Koyuncu [31], who found TA values ranging from 1.42 to 1.86%.

In general, our values of SSC, pH, and titratable acidity were in agreement with the above-mentioned studies. The calculated maturity indices (MI) of the analyzed fruits were between 8.23 and 10.66 (Table S1). Similar maturation indexes were reported by Okatan [20] (from 8.06 to 12.69) and Koyuncu [31] (7.05–11.26). The high maturity index, measured as the ratio between SSC and TA, indicates a rather sweet taste, which is in concurrence with our sensory analysis. Thus, this suggests that using these fresh fruits, with higher MI, could produce high quality fruit products.

### 3.2. Individual Sugars

Sugars are fundamental to the overall flavor profile of any fruit, as well as its nutritional properties, heavily influencing the fruits’ caloric density. Sugars are produced as the main product of photosynthesis, and are not only necessary to build up plant cell walls and provide energy, but are also essential as precursors to aroma compounds and various signaling molecules, both on a cellular and tissue level. In our study there were found significant differences (*p* ≤ 0.05) in glucose and fructose content (Table S2) among the genotypes of black mulberry, while no significant differences were evaluated for saccharose content. Generally, black and red mulberry fruits have lower total sugar content compared to white mulberries, which are much sweeter [35].

As was evaluated, black mulberry fruits contain low amounts of saccharose and much higher amounts of reduced sugars (Table S2). The proportion of glucose to fructose is quite important to overall flavor. For black mulberry genotypes in this study, we found glucose content ranging from 7.22 to 11.10 g/100 g fw, with an average value of 9.51 g/100 g. The glucose concentration found in the research of Makhoul et al. [35], which examined 11 black mulberry phenotypes cultivated in Syria, ranged from 2.21 to 14.69%, a wider scale, with an average 6.32%. Lower values of glucose content were found in 11 Greek genotypes by Roussos et al. [12], with a range of 1.83–5.85 g/100 g fw.

The fructose concentration of the twenty *M. nigra* fruit genotypes was slightly lower than their glucose content, ranging from 6.32 to 9.94 g/100 g fw, with an average of 7.85 g/100 g. The fructose content, determined by Makhoul et al. [35], of 11 Syrian phenotypes ranged from 2.25% to 11.01%, with a lower average than that of our study (4.91%). Roussos et al. [12] again detected lower fructose values than those seen in our study or in the previously mentioned study (1.88–6.25 g/100 g fw).

The lowest levels of sugars were detected for saccharose (between 1.19 and 2.28 g/100 g fw), similar to the previously mentioned research of Makhoul et al. [35], who measured sucrose concentrations ranging from 0.03% to 1.47%, with an average value 1.2%, as well as that of Roussos et al. [12], who reported values in the range of 0.03–0.16 g/100 g fw.

The calculated sweetness indices (SI), a valuation for total sweetness perception counted due to each sugar sweetness, were found to be between 23.7 and 35.3 for the 20 fruit genotypes. This indicates diversity among black mulberry fruits in terms of SI index. Higher SI values are due to higher levels of fructose in the analyzed fruits. Our results are higher than those obtained for black mulberry by Roussos et al. [12] (6.86-22.31), probably due to the different genotypes studied and the diverse microclimate.

### 3.3. Individual Organic Acids

The occurrence and amount of organic acids is an important factor influencing the organoleptic properties of fruits. Regarding organic acids in the fruits of *M. nigra*, malic, citric, oxalic, and tartaric acids are the most important. Concentration values of these acids in the fruits of the 20 analyzed black mulberry genotypes are shown in Table S3. Results indicate statistically significant differences (*p* ≤ 0.05) of malic, citric, and oxalic acid among the genotypes, though no significant differences were found for tartaric acid content.

The predominant organic acid in the fruits of the 20 black mulberry genotypes was malic acid, followed by citric, oxalic and tartaric acids, respectively. The concentration of malic acid ranged between 6.02 and11.44 g/100 g fw, with an average of 8.52 g/100 g fw. In addition, several other authors [14,17,20,31] reported the predominant organic acid in black mulberry genotypes to be malic acid. Ercisli and Orhan [14] evaluated for malic acid and reported a range of 12.3–21.8 g/100 g; Okatan [20] found that malic acid was present in amounts between 6.65 and 13.65 g/100 g fw. Citric acid, in the present study, reached only up to one third of the content of malic acid, with values ranging from 2.41 up to 4.02 g/100 g fw. Very similar data for citric acid (2.1–4.1 g/100 g fw) was determined by Ercisli and Orhan [14], with slightly higher values (2.12–7.02 g/100 g fw) found in black mulberry fruits by Okatan [20]. Oxalic and tartaric acids are present in black mulberry genotypes in similar concentrations (0.54–1.18 g/100 g fw and 0.37–1.05 g/100 g fw, respectively). This is in an agreement with the results of Okatan [20] with respect to oxalic acid and tartaric acid (0.45–1.25 g/100 g fw and 0.22–0.86 g/100 g fw, respectively).

### 3.4. Sensory Evaluation

The black mulberry genotypes were evaluated for taste, juiciness, and aroma characteristics. Four genotypes were found to have a predominantly sour taste (VC1, VC10, VC11, and VC16), four genotypes (VC2, VC8, VC17, and VC20) were predominantly sweet, and the rest (12 genotypes) were sweet–sour. In terms of juiciness, most black mulberry fruits had good juiciness (13 genotypes: VC1, VC3–6, VC9–11, VC14–16, VC18, and VC20) while the others (7 genotypes) manifested medium juiciness. Among the 20 genotypes, 16 genotypes had a very strong aroma, while four genotypes (VC4–5, VC9, and VC16) had a moderate aroma. We found great diversity in the taste, juiciness, and aroma characteristics of *Morus nigra* genotypes, which indicates a richness of the germplasm and the possibility of selecting the best cultivars for breeding studies.

### 3.5. Determination of Ascorbic Acid (Vitamin C), Total Phenolic and Total Anthocyanin Content

Ascorbic acid belongs to a group of important antioxidants with many functions contributing to optimal human health. Phenolic compounds, such as anthocyanins, are also significant antioxidants. The results of the ascorbic acid (vitamin C) content analysis, as well as the total phenolics and total anthocyanins of the 20 *M. nigra* fruit genotypes, are shown in Table 2. As was reported, all investigated parameters significantly differed among genotypes (*p* ≤ 0.05).

Ascorbic acid content was found to be 17.41–28.33 mg/100 mg fw (Table 2), with an average of 21.60 mg/100 mg fw. These values indicate a medium level of ascorbic acid, considering different types of berry fruits, such as blueberry or blackberry, that fulfil the daily intake required to prevent scurvy (10 mg/day). Previously reported vitamin C content values of black mulberry genotypes by Eyduran et al. [36] ranged lower in amount, (10.12–16.29 mg/100 g fw); a study by Okatan et al. [34] reported amounts similar to our investigated results, 18.40–23.67 mg/100 g fw. Similar results for ascorbic acid amount were also determined by several other researches for mulberry fruit species [14,16,32,37,38]. Makhoul et al. [35], evaluating Syrian black mulberry fruits, reported a wider range of ascorbic acid content than our study, from 3 to 42 mg/100 g fw, with a similar average to our value, 21.27 mg/100 g. Comparing the vitamin C values of black mulberries and white mulberries (from 2 to 16 mg/100 g fw, with an average of 3.90 mg/100 g fw), the black ones have the higher content of this vitamin.

**Figure 1:**
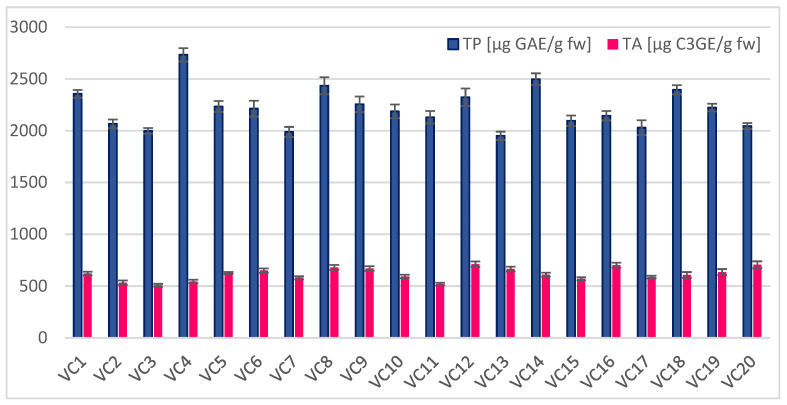
Content of total phenolics (TP) and total anthocyanins (TA) in fruits of 20 black mulberry (*M. nigra*) genotypes (VC1–VC20).

The total phenolics in the analyzed fruits ranged between 1951 and 2733 μg gallic acid equivalent/g fw (Figure 1), with an average of 2215 μg GAE/g fw. Okatan [20] found the total phenolics of 13 black mulberry genotypes sampled from the Aegean region of Turkey to be between 1874–2977 μg GAE/g fw; these values are very similar to ours. In another study by Okatan et al. [34] that analyzed eight black mulberry genotypes from eastern Turkey, they reported total phenolics ranging between 1920 and 2575 μg GAE/g fw. Ozgen et al. [17] analyzed 14 *Morus nigra* genotypes and assessed the average total phenolics to be 2737 μg GAE/g fw. Our results are comparable with the above results from different parts of Turkey. In comparison to that, Serbian research, produced by Kostic et al. [15], presented values of total phenol compounds in fresh mulberry fruits that varied from 902.6 to 1188.4 μg GAE/g fw, which is nearly half the amount of these compounds determined to be in Turkey fruits.

*M. nigra* is an anthocyanins-rich fruit species. The pigments of anthocyanins from mulberries, and other berries, show strong antioxidant effects [39]. Moreover, anthocyanin-rich berry fruits, including black mulberries, are potential functional foods that could be consumed for the prevention of some diseases. The total anthocyanins presented in investigated black mulberry genotypes ranged between 508 and 712 μg cyanidin-3-glucoside equivalent/g fresh weight (Figure 1). Ozgen et al. [17] previously reported, in *M. nigra* fruits, total anthocyanins in a wider range, from 253 to 830 μg C3GE/g fw. Okatan et al. [34] analyzed eight black mulberry genotypes from eastern Turkey and found values between 643 and 826 μg C3GE/g fw of total anthocyanins. Ercisli et al. [32], in an analysis of four black mulberry genotypes, revealed the amount of total anthocyanins to range from 674 to 787 μg C3GE/g fw. Higher amounts of anthocyanins were discovered in black mulberry genotypes by Serbian researchers [15], ranging from 1148.3 to 1286.8 μg of cyanidin-3-O-glucoside/g fw.

### 3.6. Determination of Phenolic Acids and Flavonoids

Berries are one of the best dietary sources of bioactive compounds, specifically phenolic compounds such as phenolic acids and flavonoids (anthocyanins and flavonols). These compounds are responsible for various health effects, such as stress protection, and the prevention of several disorders (inflammation, cardiovascular diseases, and lower risk of some cancers) [40].

Table 2 and Table 3 show the main phenolic acid and flavonoid contents of the fruits of the 20 black mulberry genotypes, indicating significant differences among phenolic acids, namely chlorogenic, gallic, caffeic acid, and ellagic acid, as well as flavonoids rutin, quercetin, catechin, at *p* ≤ 0.05.

The major phenolic acid in the black mulberry fruit samples was chlorogenic acid, which was determined to be in the range of 51.3–90.8 mg/100 g fw. Less than half amount of the major acid was analyzed for gallic acid (22.40–38.56 mg/100 g fw), followed by caffeic acid content (7.22–15.30 mg/100 g fw), and finally ellagic acid (2.15–6.32 mg/100 g fw). Okatan [20] reported the highest concentration in the fruits of the same species to be chlorogenic acid (43–97 mg/100 g fw), followed by gallic acid (21.83–40.90 mg/100 g fw). Some authors previously found chlorogenic acid to be one of the main phenolic compounds in black mulberry fruits [36,41,42].

As for important flavonoids in black mulberry fruits, we analyzed rutin, quercetin, and catechin (Table 3. Rutin, quercetin-3-O-rutinoside, ranging from 47.10 to 97.20 mg/100 g fw, was determined to be the predominant flavonoid. Several studies defined rutin as the most relevant flavonoid in black mulberry fruits [36,41,42]. In addition, Okatan [20] reported the highest concentration of rutin (92–133 mg/100 g) in the fruits of the same species. Lower flavonoid amounts in the fruits analyzed in the present study were evaluated for quercetin (3.11–9.78 mg/100 g fw) and catechin (3.15–9.40 mg/100 g fw).

Concerning chlorogenic and gallic acids, as well as rutin, there was found a strong positive association with antioxidant activity evaluated by the FRAP assay (r = 0.829; 0.762; 0.745, respectively).

### 3.7. Determination of Antioxidant Activity

**Figure 2:**
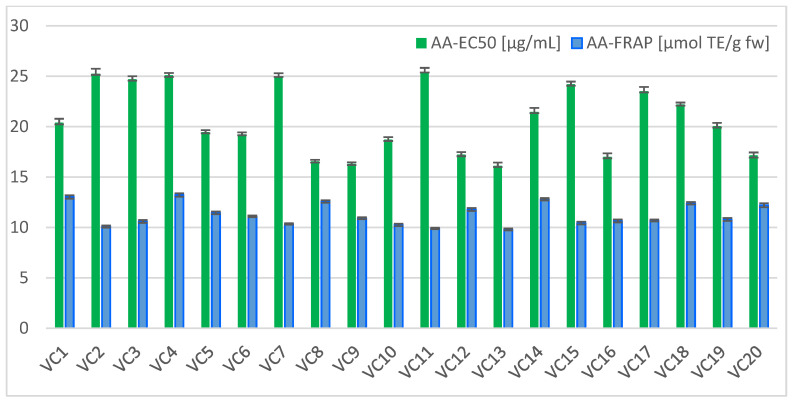
Antioxidant activity (AA) by DPPH (EC_50_) and FRAP assays in fruits of 20 black mulberry (*M. nigra*) genotypes (VC1–VC20).

To assess the antioxidant potential of plant products, which contain a great amount of polar compounds, assays such as the DPPH and FRAP tests are the most common and suitable methods. The antioxidant activity (AA) results of the twenty analyzed *M. nigra* fruit genotypes, determined by DPPH and FRAP assays, are shown in Figure 2. Both investigated parameters significantly differed among genotypes (*p* ≤ 0.05).

Antioxidant activity in the DPPH assay was expressed as half of the maximal effective concentration (EC_50_), where the lowest value of EC_50_ denotes the highest antioxidant activity. The EC_50_ values found in this work ranged between 17.22–25.45 μg/mL; therefore, the highest AA was in the black mulberry sample with the lowest value of this parameter. Similar AA results, using EC_50_ concentration, were reported Okatan [20]. In his study the values of antioxidant activity in fresh fruits, for 13 black mulberry genotypes from the Aegean region of Turkey, were evaluated to be between 16.87 and 26.80 μg/mL. Okatan et al. [34] analyzed eight black mulberry genotypes and assessed antioxidant activity, by EC_50_ values, in the range of 18.24–23.18 μg/mL.

AA was also assessed by the reaction of electron donating antioxidants with the reduction of colorless ferric complex (Fe^3+^) to colored Fe^2+^ complex (FRAP assay). Antioxidant activity, measured by the FRAP test, of *M. nigra* genotypes for the 20 fruit samples was determined to be in the range of 9.80 to 13.22 μmol TE/g fw. In comparison with the results of other berry fruits [43], such as the best source, bilberry (82.3 μmol TE/g), followed by black currant (73.5 μmol TE/g), blackberry, elderberry, strawberry, red currant (17.8 μmol TE/g), and gooseberries (14.5 μmol TE/g), black mulberries can be considered a species with lower antioxidant activity. In comparison with some other fruits, such as orange (11.4 μmol TE/g), plum, kiwi, lemon, and apple (2.9 μmol TE/g), black mulberries belong to types with higher antioxidant activity.

To characterize the relationship between the amount of antioxidants, such as ascorbic acid, total phenolics, anthocyanins, and antioxidant activity, we calculated the correlation coefficients. Antioxidant activity, determined by a DPPH assay, was strongly negatively associated with anthocyanin content (r = −0.918); this is in an agreement with the fact that low values on a DPPH test denote high AA. Ascorbic acid had only a weak correlation with AA results, measured by the DPPH assay (r = 0.090). For antioxidant activity, evaluated by the FRAP assay, there was found a strong positive association with phenolics (r = 0.818), and only a weak correlation between AA results and ascorbic acid. Generally, this indicates that anthocyanins and phenolics are important contributors to antioxidant activity. In addition, Kamiloglu et al. [41] reported a relationship between total phenolics and antioxidant capacity.

### 3.8. Principal Coordinate Analysis (PCoA)

For the principal coordinate analysis (PCoA), the searched morphological and biochemical parameters were considered. As a morphological parameter, fruit weight was used. For biochemical parameters, SSC, pH, titratable acidity, maturity index, individual sugars (fructose, glucose, and saccharose), organic acids (malic, citric, oxalic, and tartaric), ascorbic acid, total phenolic and total anthocyanin contents, antioxidant activity (DPPH and FRAP tests), and phenolic acids and flavonoids (chlorogenic acid, gallic acid, caffeic acid, ellagic acid, rutin, quercetin, and catechin) were used.

**Figure 3:**
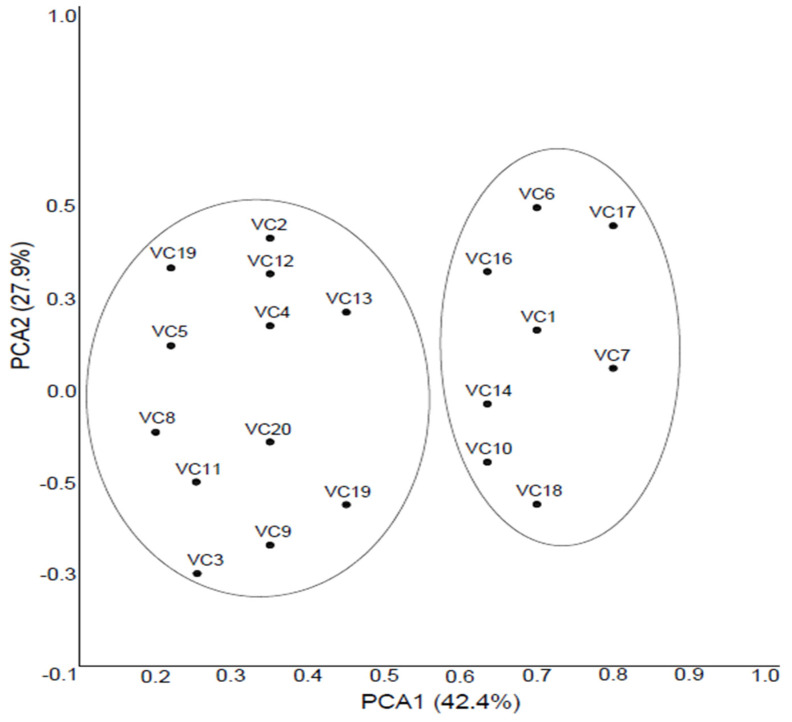
PCoA analysis of 20 black mulberry genotypes.

The grouping of each genotype was based on the proportion of total diversity. The contribution of the first three principal coordinates in the Principal Coordinate Analysis (PCoA) represented 80.5% of the variance. The components with the largest proportion of diversity were component 1 (PCA1) and component 2 (PCA2), which reached 70.3% (42.4% and 27.9%, respectively) of the overall variance; thus, the grouping was made based on these components. The third principal coordinate explained 10.2% of the overall variance. Black mulberry genotypes were, therefore, partitioned into two distinct groups: PCoA Group 1 included 12 samples and Group 2 included eight samples, respectively (Figure 3).

Characters that affect genetic diversity in the principal components are determined by the value of eigenvector. The characteristic vector value > 0.5 indicates that the character affected the diversity. The first group was characterized by samples with high SSC, total phenolic, and antioxidant activity (DPPH and FRAP assays). The high values of these measured parameters were detected especially for three genotypes of *M. nigra* (VC4, VC8, and VC20) located in first group of samples (Figure 3). The mentioned samples were therefore selected as genotypes with good quality black mulberry fruits (sweet taste with high antioxidant characteristics) and recommended for production in Turkey.

The PCoA revealed useful information on the characterization and comparison of black mulberry genotypes based on biochemical and morphological data. Substantial dispersion of black mulberry genotypes in the PCoA plot suggests relative diversity in fruit composition, which can make them attractive for future breeding programs.

## 4. Conclusions

Fruit quality is the most important factor for both consumers and producers. The results of this study show significant differences between black mulberry genotypes in their phytochemical and antioxidant characteristics. The high antioxidant qualities evaluated in black mulberry fruits underline their nutritive and phytomedicinal potential. High sweetness and antioxidant parameters (content of phenolics, anthocyanins, ascorbic acid, chlorogenic acid, and rutin), as well as high antioxidant activity, were determined for several genotypes (VC4, VC8, VC12, VC14, VC16, and VC20). Regarding the best values of phytochemical and antioxidant characteristics, three genotypes of *M. nigra* (VC4, VC8, and VC20) were selected to be recommended for production in Turkey, because there is an increased demand for quality fruits of black mulberry (*Morus nigra*) with lower ecological burden without chemical products, as well as without diseases and pests. The results thus highlight the potential for the exploitation of local black mulberry genotypes through crop selection and breeding programs.

## Figures and Tables

**Table 1:** Characteristics of analyzed *M. nigra* fruits; average fruit weight and fruit shape index.

Genotype	Location in Turkey	Altitude [m]	Fruit Weight [g]	Fruit Shape Index
VC1	Ardanuc	548	4.88 ± 0.21 ^b,c^	0.76 ± 0.08 ^a,b^
VC2	Ovacik	1141	4.69 ± 0.17 ^b,c^	0.80 ± 0.09 ^a,b^
VC3	Harmanli	711	4.56 ± 0.20 ^b,c^	0.71 ± 0.06 ^a,b^
VC4	Ciritduzu	1153	5.28 ± 0.32 ^a,b^	0.74 ± 0.07 ^a,b^
VC5	Tepeduzu	1134	5.09 ± 0.24 ^a,b^	0.69 ± 0.05 ^b^
VC6	Ortulu	869	4.37 ± 0.13 ^c^	0.75 ± 0.09 ^a,b^
VC7	Uzumlu	1065	4.48 ± 0.10 ^b,c^	0.78 ± 0.06 ^a,b^
VC8	Sakarya	1200	4.71 ± 0.15 ^b,c^	0.81 ± 0.11 ^a^
VC9	Yolustu	1002	5.55 ± 0.22 ^a^	0.73 ± 0.08 ^a,b^
VC10	Torbali	723	5.14 ± 0.19 ^a,b^	0.70 ± 0.06 ^a,b^
VC11	Eksinar	540	5.42 ± 0.30 ^a,b^	0.74 ± 0.08 ^a,b^
VC12	Dalkirmaz	1031	4.62 ± 0.18 ^b,c^	0.76 ± 0.10 ^a,b^
VC13	Susuz	982	5.40 ± 0.16 ^a,b^	0.78 ± 0.11 ^a,b^
VC14	Ciftlik	1160	5.02 ± 0.32 ^b^	0.74 ± 0.06 ^a,b^
VC15	Ovacik	1145	5.31 ± 0.27 ^a,b^	0.70 ± 0.04 ^a,b^
VC16	Cayagzi	740	4.80 ± 0.12 ^b,c^	0.73 ± 0.08 ^a,b^
VC17	Ciralar	1218	5.35 ± 0.22 ^a,b^	0.75 ± 0.09 ^a,b^
VC18	Cayagzi	932	5.20 ± 0.24 ^a,b^	0.71 ± 0.05 ^a,b^
VC19	Dutlu	1119	5.07 ± 0.19 ^a,b^	0.69 ± 0.06 ^a,b^
VC20	Bereket	1188	4.48 ± 0.16 ^b,c^	0.74 ± 0.08 ^a,b^

Different letters in the last column indicate significantly different values at *p* ≤ 0.05.

**Table 2:** Content of ascorbic and phenolic acids in fruits of *M. nigra* genotypes.

Genotype	Ascorbic Acid [g/100 g fw]	Chlorogenic Acid [g/100 g fw]	Gallic Acid [g/100 g fw]	Caffeic Acid [g/100 g fw]	Ellagic Acid [g/100 g fw]
VC1	20.47 ± 0.44 ^c,d^	80.5 ± 1.35 ^b^	35.4 ± 0.9 ^a,b^	14.00 ± 0.2 ^a,b^	4.40 ± 0.2 ^a,b^
VC2	25.48 ± 0.77 ^a,b^	59.6 ± 1.44 ^d,e^	24.2 ± 0.8 ^b,c^	8.45 ± 0.3 ^a,b^	3.89 ± 0.1 ^a,b^
VC3	28.3 ± 1.10 ^a^	60.3 ± 1.04 ^d,e^	28.4 ± 0.8 ^c,d^	11.08 ± 0.1 ^a,b^	6.32 ± 0.2 ^a^
VC4	25.40 ± 0.80 ^b^	85.4 ± 1.84 ^a,b^	37.2 ± 1.0 ^a,b^	14.84 ± 0.5 ^a,b^	3.55 ± 0.1 ^a,b^
VC5	23.66 ± 0.61 ^b,c^	67.3 ± 1.14 ^c,d^	31.5 ± 0.9 ^a,b^	7.22 ± 0.4 ^b^	2.98 ± 0.1 ^a,b^
VC6	20.70 ± 0.43 ^c,d^	71.6 ± 1.00 ^b,c^	34.1 ± 0.5 ^a,b^	11.04 ± 0.3 ^a,b^	2.15 ± 0.1 ^b^
VC7	19.36 ± 0.37 ^d,e^	62.4 ± 0.78 ^c,d^	30.5 ± 0.5 ^b,c^	12.06 ± 0.3 ^a,b^	4.70 ± 0.2 ^a,b^
VC8	21.90 ± 0.42 ^c,d^	82.6 ± 3.11 ^a,b^	38.5 ± 0.8 ^a^	13.90 ± 0.3 ^a,b^	5.20 ± 0.2 ^a,b^
VC9	26.40 ± 0.80 ^a,b^	70.7 ± 2.44 ^c^	30.6 ± 0.4 ^b^	14.50 ± 0.4 ^a,b^	5.80 ± 0.2 ^a,b^
VC10	17.41 ± 0.35 ^e^	69.5 ± 1.60 ^c,d^	31.2 ± 0.6 ^a,b^	10.65 ± 0.3 ^a,b^	4.78 ± 0.1 ^a,b^
VC11	22.56 ± 0.58 ^c^	66.6 ± 1.54 ^c,d^	29.0 ± 0.5 ^b,c^	12.00 ± 0.5 ^a,b^	4.00 ± 0.1 ^a,b^
VC12	19.02 ± 0.50 ^d,e^	74.3 ± 1.76 ^b,c^	35.3 ± 1.1 ^a,b^	15.30 ± 0.2 ^a^	5.10 ± 0.2 ^a,b^
VC13	24.80 ± 0.43 ^b,c^	51.3 ± 1.20 ^e^	22.4 ± 0.4 ^c^	14.10 ± 0.2 ^a,b^	4.90 ± 0.2 ^a,b^
VC14	19.50 ± 0.62 ^d,e^	90.8 ± 2.67 ^a^	37.9 ± 1.2 ^a,b^	13.40 ± 0.4 ^a,b^	3.03 ± 0.1 ^a,b^
VC15	18.80 ± 0.54 ^d,e^	60.7 ± 1.05 ^d^	29.6 ± 1.0 ^b,c^	11.60 ± 0.5 ^a,b^	5.90 ± 0.2 ^a,b^
VC16	20.11 ± 0.29 ^c,d^	70.7 ± 1.55 ^c^	31.5 ± 1.1 ^a,b^	9.44 ± 0.3 ^a,b^	3.25 ± 0.1 ^a,b^
VC17	18.30 ± 0.66 ^d,e^	65.4 ± 1.62 ^c,d^	26.5 ± 0.7 ^b,c^	12.44 ± 0.7 ^a,b^	5.40 ± 0.2 ^a,b^
VC18	18.60 ± 0.55 ^d,e^	73.4 ± 1.04 ^b,c^	32.8 ± 1.2 ^a,b^	13.36 ± 0.5 ^a,b^	6.09 ± 0.2 ^a,b^
VC19	19.87 ± 0.50 ^d^	79.6 ± 1.84 ^b,c^	33.8 ± 1.0 ^a,b^	13.02 ± 0.9 ^a,b^	3.70 ± 0.1 ^a,b^
VC20	21.27 ± 0.63 ^c,d^	72.5 ± 1.42 ^b,c^	28.2 ± 0.7 ^b,c^	11.00 ± 0.8 ^a,b^	4.90 ± 0.1 ^a,b^

Different letters in columns indicate significantly different values at *p* ≤ 0.05.

**Table 3:** Content of flavonoids in fruits of *M. nigra* genotypes.

Genotype	Rutin [mg/100 g fw]	Quercetin [mg/100 g fw]	Catechin [mg/100 g fw]
VC1	85.4±1.74 ^b^	5.50 ± 0.1 ^ab^	7.10 ± 0.1 ^b^
VC2	67.7 ± 1.45 ^cd^	4.40 ± 0.2 ^ab^	4.10 ± 0.2 ^cd^
VC3	62.1 ± 0.95 ^cd^	8.02 ± 0.4 ^ab^	9.40 ± 0.3 ^a^
VC4	97.2 ± 3.31 ^a^	3.88 ± 0.3 ^ab^	3.15 ± 0.2 ^d^
VC5	64.3 ± 2.20 ^cd^	3.11 ± 0.1 ^b^	4.35 ± 0.2 ^cd^
VC6	74.0 ± 1.10 ^bc^	4.90 ± 0.2 ^ab^	5.40 ± 0.1 ^c^
VC7	58.6 ± 1.07 ^d^	7.70 ± 0.5 ^ab^	4.50 ± 0.1 ^cd^
VC8	94.3 ± 2.88 ^ab^	7.22 ± 0.5 ^ab^	5.30 ± 0.2 ^cd^
VC9	72.4 ± 2.51 ^bc^	4.90 ± 0.3 ^ab^	4.00 ± 0.1 ^cd^
VC10	73.8 ± 3.12 ^bc^	6.23 ± 0.3 ^ab^	4.80 ± 0.2 ^cd^
VC11	71.4 ± 2.56 ^c^	6.80 ± 0.2 ^ab^	5.20 ± 0.2 ^cd^
VC12	75.6 ± 1.89 ^bc^	5.89 ± 0.2 ^ab^	4.65 ± 0.1 ^cd^
VC13	47.1 ± 0.77 ^de^	6.80 ± 0.1 ^ab^	6.40 ± 0.3 ^bc^
VC14	96.7 ± 2.45 ^ab^	4.90 ± 0.2 ^ab^	4.00 ± 0.1 ^cd^
VC15	69.6 ± 1.88 ^cd^	7.02 ± 0.4 ^ab^	8.07 ± 0.4 ^ab^
VC16	77.3 ± 2.05 ^bc^	7.56 ± 0.2 ^ab^	6.80 ± 0.2 ^bc^
VC17	69.3 ± 1.20 ^cd^	9.78 ± 0.5 ^a^	8.00 ± 0.2 ^ab^
VC18	79.6 ± 3.13 ^bc^	7.70 ± 0.2 ^ab^	5.32 ± 0.1 ^cd^
VC19	88.6 ± 2.90 ^ab^	8.08 ± 0.3 ^ab^	8.20 ± 0.3 ^ab^
VC20	70.4 ± 1.16 ^cd^	4.08 ± 0.1 ^ab^	4.50 ± 0.1 ^cd^

Different letters in columns indicate significantly different values at *p* ≤ 0.05.

## Data Availability

The data are available from the author Sezai Ercisli upon request.

## Supplementary Materials

The following supporting information can be downloaded at: https://www.mdpi.com/article/10.3390/antiox11071339/s1, Table S1: Soluble solid content (SSC), pH, titratable acidity (TA), and maturity index (MI) of M. nigra geno-types.; Table S2: Content of Glu (glucose), Fru (fructose), Sacch (saccharose), and Sweetness Index (SI) in fruits of M. nigra genotypes.; Table S3: Content of organic acids in fruits of M. nigra genotypes.
