# Supplementary material for: Diversity of Phytochemical and Antioxidant Characteristics of Black Mulberry (Morus nigra L.) Fruits from Turkey
Source: Antioxidants (Basel). 2022 Jul 8;11(7):1339. doi: 10.3390/antiox11071339 (PMC9311561; doi:10.3390/antiox11071339)
Supplement: Supplementary file 1 [file antioxidants-11-01339-s001.zip › antioxidants-1795754-supplementary.pdf]

antioxidants

MDPI

Table S1. Soluble solid content (SSC), pH, titratable acidity (TA), and maturity index (MI) of *M. nigra* genotypes.

Genotype	SSC [%]	pH	TA [%]	MI
VC1	14.07±0.67[de]	3.60±0.08[ab]	1.49±0.02[NS]	9.44±0.28[ab]
VC2	16.33±1.04[bc]	3.82±0.12[ab]	1.55±0.03	10.53±0.19[ab]
VC3	14.41±0.08[de]	3.70±0.07[ab]	1.38±0.01	10.44±0.09[ab]
VC4	16.57±0.77[bc]	3.79±0.05[ab]	1.82±0.04	9.10±0.12[ab]
VC5	16.30±0.60[bc]	3.98±0.10[ab]	1.70±0.06	9.59±0.17[ab]
VC6	15.15±0.74[cd]	3.55±0.09[b]	1.80±0.08	8.42±0.10[ab]
VC7	16.10±0.85[bc]	3.71±0.07[ab]	1.70±0.05	9.47±0.13[ab]
VC8	17.20±0.55[ab]	3.86±0.10[ab]	1.74±0.04	9.88±0.15[ab]
VC9	15.67±0.49[cd]	3.65±0.04[ab]	1.68±0.03	9.33±0.17[ab]
VC10	14.56±0.40[de]	3.77±0.05[ab]	1.77±0.07	8.23±0.09[b]
VC11	13.36±0.36[ef]	4.12±0.10[a]	1.48±0.06	9.03±0.12[ab]
VC12	15.83±0.66[c]	4.05±0.10[ab]	1.59±0.06	9.96±0.10[ab]
VC13	15.40±0.54[cd]	3.90±0.09[ab]	1.87±0.08	8.24±0.14[b]
VC14	16.96±0.39[b]	3.75±0.07[ab]	1.75±0.05	9.69±0.11[ab]
VC15	16.50±0.70[bc]	3.89±0.05[ab]	1.72±0.05	9.59±0.15[ab]
VC16	14.83±0.52[d]	3.68±0.07[ab]	1.55±0.02	9.56±0.07[b]
VC17	17.95±1.07[a]	3.95±0.06[ab]	1.80±0.10	9.97±0.14[ab]
VC18	15.30±0.61[cd]	4.03±0.08[ab]	1.62±0.05	9.44±0.10[ab]
VC19	16.22±0.49[bc]	3.97±0.10[ab]	1.56±0.04	10.39±0.08[ab]
VC20	14.07±0.67[de]	3.94±0.08[ab]	1.60±0.04	10.66±0.09[a]

[NS]: Non significant; Different letters in columns indicate significantly different values at $p \leq 0.05$.

Table S2. Content of Glu (glucose), Fru (fructose), Sacch (saccharose), and Sweetness Index (SI) in fruits of *M. nigra* genotypes.

Genotype	Glu [g/100 g fw]	Fru [g/100 g fw]	Sacch [g/100 g fw]	SI
VC1	7.56±0.4[de]	6.60±0.2[ab]	1.60±0.02[NS]	24.9±1.0[bc]
VC2	10.34±0.5[ab]	8.82±0.4[ab]	1.83±0.03	33.1±1.2[ab]
VC3	8.04±0.3[de]	7.56±0.1[ab]	1.21±0.02	27.4±0.9[bc]
VC4	10.20±0.5[ab]	8.70±0.2[ab]	1.86±0.04	32.7±1.1[ab]
VC5	10.04±0.5[bc]	8.56±0.3[ab]	1.75±0.05	32.2±1.0[ab]
VC6	9.18±0.3[c]	7.28±0.3[ab]	1.33±0.04	27.7±0.9[bc]
VC7	9.90±0.3[bc]	8.40±0.3[ab]	2.00±0.06	31.9±1.1[ab]
VC8	10.84±0.4[ab]	9.94±0.4[a]	1.19±0.02	35.3±1.0[a]
VC9	10.10±0.5[b]	7.13±0.2[ab]	1.82±0.03	29.0±0.7[b]
VC10	8.13±0.2[d]	6.44±0.2[ab]	1.50±0.04	25.0±1.0[bc]
VC11	7.22±0.3[e]	6.32±0.2[b]	1.43±0.04	23.7±0.7[c]
VC12	9.90±0.3[bc]	7.44±0.2[ab]	1.40±0.05	28.9±0.8[b]
VC13	10.34±0.4[ab]	7.70±0.3[ab]	2.28±0.06	31.1±1.0[ab]
VC14	9.70±0.3[bc]	8.40±0.3[ab]	1.84±0.04	31.5±0.6[ab]
VC15	9.46±0.2[bc]	8.22±0.4[ab]	1.66±0.03	30.6±0.8[ab]
VC16	7.41±0.1[de]	6.55±0.2[ab]	1.33±0.03	24.3±0.6[bc]
VC17	11.10±0.4[a]	8.78±0.2[ab]	1.90±0.03	33.9±1.0[ab]
VC18	9.77±0.2[bc]	7.50±0.3[ab]	1.27±0.04	28.7±0.6[bc]
VC19	10.24±0.3[ab]	7.75±0.2[ab]	1.95±0.04	30.7±0.8[ab]

VC20	10.70±0.4[ab]	8.90±0.3[ab]	1.75±0.05	33.5±0.9[ab]

[NS]: Non-significant; Different letters in columns indicate significantly different values at $p \leq 0.05$.

Table S3. Content of organic acids in fruits of *M. nigra* genotypes.

Genotype	Malic acid [g/100 g fw]	Citric acid [g/100 g fw]	Oxalic acid [g/100 g fw]	Tartaric acid [g/100 g fw]
VC1	6.75±0.3[bc]	3.87±0.09[ab]	0.54±0.03[b]	0.37±0.03[NS]
VC2	7.02±0.2[bc]	3.44±0.07[ab]	0.70±0.02[ab]	0.60±0.02
VC3	6.02±0.1[c]	2.78±0.12[ab]	0.66±0.03[ab]	0.55±0.04
VC4	9.23±0.4[ab]	2.97±0.08[ab]	1.18±0.02[a]	0.74±0.03
VC5	7.10±0.3[bc]	2.41±0.10[b]	0.58±0.01[ab]	0.78±0.02
VC6	8.66±0.4[bc]	3.13±0.13[ab]	1.07±0.05[ab]	0.80±0.01
VC7	8.40±0.4[bc]	3.56±0.09[ab]	0.90±0.06[ab]	0.84±0.02
VC8	8.89±0.5[bc]	3.83±0.07[ab]	0.99±0.03[ab]	1.05±0.01
VC9	9.00±0.5[b]	3.72±0.05[ab]	0.75±0.02[ab]	0.61±0.02
VC10	10.80±0.8[ab]	3.40±0.04[ab]	0.90±0.05[ab]	0.77±0.03
VC11	7.93±0.3[bc]	3.03±0.03[ab]	0.61±0.06[ab]	0.70±0.02
VC12	8.74±0.5[bc]	3.60±0.07[ab]	0.85±0.03[ab]	0.68±0.02
VC13	11.44±0.6[a]	3.65±0.08[ab]	1.03±0.04[ab]	0.90±0.03
VC14	11.20±0.6[ab]	4.02±0.05[a]	0.80±0.02[ab]	0.80±0.02
VC15	9.04±0.5[c]	2.80±0.07[ab]	1.10±0.02[ab]	0.75±0.02
VC16	6.92±0.4[bc]	2.85±0.05[ab]	0.59±0.03[ab]	0.44±0.01
VC17	10.38±0.5[ab]	2.50±0.07[ab]	1.05±0.04[ab]	0.95±0.03
VC18	7.10±0.2[bc]	2.70±0.09[ab]	0.64±0.05[ab]	0.48±0.01
VC19	7.77±0.3bc	2.90±0.10[ab]	0.67±0.05[ab]	0.52±0.01
VC20	8.06±0.4[bc]	3.35±0.11[ab]	0.80±0.04[ab]	0.66±0.02

[NS]: Non-significant; Different letters in columns indicate significantly different values at $p \leq 0.05$.
